# Oligopeptide derived from solid-state fermented cottonseed meal significantly affect the immunomodulatory in BALB/c mice treated with cyclophosphamide

**DOI:** 10.1007/s10068-018-0414-1

**Published:** 2018-06-20

**Authors:** Jiancheng Liu, Hong Sun, Cunxi Nie, Wenxia Ge, Yongqiang Wang, Wenju Zhang

**Affiliations:** 1 College of Animal Science and Technology, Shihezi University, North Street 4, 832000 Shihezi, China; 2 Institute of Plant Protection and Microbiology, Zhejiang Academy of Agriculture Sciences, Hangzhou, China

**Keywords:** Cottonseed meal, Solid-state fermentation, Oligopeptide, Cyclophosphamide, Immunomodulatory

## Abstract

In this study, the immunomodulatory activity of oligopeptide (CP) derived from solid-state fermented cottonseed meal were investigated in immunosuppressed BALB/c mice models by treatment with cyclophosphamide (CY). Results indicated that oligopeptide increased the thymus and spleen indices of CY-treated mice. The count of plague forming cells (PFC) and the content of half serum hemolysis (HC_50_) in immunosuppressive mice were restored to the normal level in CP-10 and CP-20 groups while the cytokines interleukin (IL)-2, IL-6, and tumor necrosis factor alpha (TNF-α) were increased significantly in CP-20 group. Similar increasing the immunoglobulin of IgG and IgM content in the serum of CP-10 group were also observed. These findings indicated that oligopeptide derived from solid-state fermented cottonseed meal had a strong immune-enhancing activity as well as a protective effect against immunosuppression induced by cyclophosphamide in mice.

## Introduction

According to the modern protein nutrition theory, protein is absorbed in animal digestive tract and could be divided into two forms, free amino acids and oligopeptide. Particularly, oligopeptide is the main digestive product of protein and an important form for absorption (Gilbert et al., 2008). The bioactive peptides derived from natural food proteins have attracted much attention in recently years and are considered to perform physiological activities in the major body systems (Maestri et al., 2016). An array of biological activities of peptides derived from various food sources, including antioxidative (Bernardini et al., 2011), antihypertensive (Contreras et al., 2011), antidiabetic (Shen et al., 2012) and immunomodulatory activities (Qian et al., 2011) has been reported.

In recent years, an abundance of studies have focused on the immune regulation activity of peptides from animal and plant proteins (Bhat et al., 2015; Maestri et al., 2016). Studies have shown that peptides derived from food protein hydrolysates in the body can not only improve animal productivity, but also promote animal immunity. Some peptides, which were called immunomodulatory peptides, can promote the immune functions by controlling the humoral immunity and cell-mediated immune response (Haney and Hancock, 2013). Immunomodulatory peptides have attracted much attention because they can enhance the immunity of organisms, stimulate the proliferation of lymphocytes, increase the phagocytic activity of macrophages as well as defense against pathogens in organisms, promote the antibody production and regulate the transformation of T and B cells, and stimulate the secret of cytokine and immunoglobulin (Mallet et al., 2017). Recently, various bioactive peptides derived from enzymatic hydrolysis of soybean (Mallet et al., 2017), chickpea (Xue et al., 2015), rapeseed (Wang et al., 2016), wheat (Kawaguchi et al., 2017), rice (Taniguchi et al., 2017) has been shown interesting immunomodulatory ability.

In the present study, the oligopeptide derived from solid-state fermentation cottonseed meal was isolated and purified, and the basic chemical characterization was investigated. Particularly, the immunomodulatory effect of oligopeptide was evaluated in immunosuppressed mice induced by cyclophosphamide.

## Materials and methods

### Materials

Defatted cottonseed meal was purchased from Tiankang Oilseed Processing Co., Ltd. (Xinjiang, China); *Bacillus subtilis* (CICC1201) and *Saccharomyces cerevisiae* (CICC 1001) were purchased from China General Microbiological Culture Collection (Beijing, China); Cyclophosphamide was purchased from Heng rui Pharmaceutical Co., Ltd. (Jiangsu, China); Enzyme-linked immunosorbent assay (ELISA) kits of interleukin (IL)-2, IL-6, tumor necrosis factor (TNF-α), and IgG and IgM were purchased from Cheng Lin Biotechnology Co., Ltd. (Beijing, China); Roswell park memorial institute medium (RPMI 1640) and sheep red blood cells (SRBCs) were purchased from Sangon Biotech Co., Ltd. (Shanghai, China); Guinea pig serum were made in our laboratory according to the instruction (Li et al., 2011). All other chemicals, and reagents used in this study were of analytical grade and were purchased from Yongsheng Fine Chemical Co., Ltd. (Tianjin, China).

### Solid-state fermentation of cottonseed meal

The solid-state fermentation of cottonseed meal was prompted using the method of Sun et al. (2015), with some modifications. Briefly, 100 g of cottonseed meal was mixed thoroughly with 60 mL of distilled water (containing 3 g sucrose) in a screw-capped glass bottle (500 mL), and sterilized at 121 °C for 20 min. Then after cooling this to room temperature, a 2% mixture of bacterium (*Bacillus subtilis: Saccharomyces cerevisiae*; volume rate: 1:1) was added and the solution was incubated at 30 °C for 72 h, with shaking the fermentation every 24 h. After fermentation, the samples were dried at 45 °C for 24 h and mashed though a 40 mesh sieve, then stored at − 80 °C for further study.

### Isolation of oligopeptide and their molecular weight distribution

The extraction of oligopeptide was done according to the methods of Sun et al. (2015), with some modification. Briefly, 10 g of fermented cottonseed meal was mixed with 150 mL distilled water and shake slowly for 30 min. Then, the mixtures were centrifuged at 4000×*g* at 4 °C for 20 min and the supernatant were filtered through a nitrocellulose membrane (0.22 μm). The supernatant was filtered with an ultrafiltration centrifuge tube (molecular weight ≤ 3000 Da), and after desalting with dextran G-25 gel column, the mixture was concentrated by rotary evaporation (RE-5298A, Shanghai) at 40 °C and vacuum-dried to obtain the oligopeptide. Then, the weight distributions of oligopeptide were determined in a Waters model 2695 Separations Module (Waters, Corp., Milford, MA, USA) using a TSKgelG2000SWXL column (300 mm × 7.8 mm). The mobile phase was a mixture solution (acetonitrile: water: trifluoroacetic acid; volume rate: 20:80:0.1) at flow rate of 0.5 mL/min. Distribution of the oligopeptide’ molecular weight was detected using a ultraviolet–visible photodiode array detector (Waters 2489 UV/Vis Detector; Waters Corp., Milford, MA, USA) at a wavelength of 200 nm.

### Determination of the proximate composition of solid-fermented cottonseed meal

The crude contents of protein, fat, fiber, ash, free gossypol, and amino acids were determined according to the National Standard of the People’s Republic of China, as described by Tian et al. (2016). Briefly, the crude protein content was evaluated with a Kjeldahl apparatus (KDN-1; Shanghai Ray Magnetic Co., Ltd., China) using a nitrogen-to-protein conversion factor of 6.25 (GB5009.5-2010). The ash (%) content was determined by weighing oligopeptide before and after heat treatment in a muffle (SX2-4-10F; Yiheng Co., Ltd., China) at 550 °C for 4 h (GB5009.4-2010). The crude fat content was determined by weighing samples and extracting the crude fat with hexane with a Soxhlet (SZC-D; Xianjian Instruments Co., Ltd., Shanghai) apparatus (GB/T 14,772-2008). To determine the crude fiber content, the solid-fermented cottonseed meal was boiled in 0.255 mol/L sulfuric acid for 30 min, filtered, washed, boiled in 0.313 mol/L sodium hydroxide, filtered, and washed again, and dried at 130 ± 2 °C for 2 h (GB/T 5009.10-2003). The free gossypols were measured with a spectrophotometer at the absorption wavelength of 400 nm (GB/T13086-1991). And amino acids were determined with an automatic amino acid analyzer (835-50; Hitachi, Tokyo, Japan) according to provided introductions.

### Animals and experimental design

BALB/c mice (6 weeks old, female, weight of 18–22 g) were purchased from the Shihezi University Laboratory Animal Center (Xingjiang, China), the mice were housed in a rodent facility at 20 °C with a 12 h light–dark cycle and could take water and food freely. All procedures involving mice and their care were carried out in accordance with the guidelines of the ethics committee of Shihezi University in Shihezi, China (A2014-069-01). The mice were randomly divided into 5 groups consisting of 18 mice each. All mice were allowed 1 week to adapt to their environment prior to undergoing experiments. The experimental period for each group was 10 days. One group of healthy mice was used as a normal control (NC) group and were treated once daily with physiological saline for 10 days. For days 1–3, the other 4 groups of mice were given cyclophosphamide at 80 mg/kg body weight (BW)/d via intraperitoneal injection. For days 4–10, the mice were administered as follows: model control group (CY), physiological saline was administered via gavage in 0.25 mL; three oligopeptide groups (CP-5, CP-10 and CP-20), at 5, 10, and 20 mg/mL oligopeptide were administered via gavage in 0.25 mL solutions. Twenty-four hours after the last drug administration, the mice were weighed and sacrificed by cervical dislocation and various immune indices were measured.

### Immune organ indices

The immune organs, spleen and thymus of each animal of all experimental groups were collected following sacrifice of the animal. The spleen and thymus of each animal were weighed upon collection. Subsequently, the thymus index and spleen index, respectively, were calculated according to the following equations:

$${\text{Thymus index}} = \frac{\text{Thymus weight}}{\text{Mouse weight}} \quad {\text{Spleen index}} = \frac{\text{Spleen weight}}{\text{Mouse weight}}$$

### Determination of plague forming cell content (PFC)

The effects of cyclophosphamide on the plague forming cell were determined by the method of Raj and Gothandam (2015) with slight modification. Six mice randomly selected from each group were sacrificed and their spleens were aseptically collected. Single-cell spleen suspensions were pooled in serum-free RPMI-1640 medium by filtering the suspension through sieve mesh with the aid of a glass homogenizer to exert gentle pressure on the spleen fragments. Samples were washed twice with serum-free RPMI-1640 medium and cells were suspended at a concentration of 1 × 10^6^ cells/mL in the same medium. Next, 0.5 mL of spleen cells (1 × 10^6^ cells/mL), 1 mL of 0.2% SRBCs, and 1 mL of 10% guinea pig serum were mixed into a glass Petri plate and incubated for 1 h at 37 °C and at 5% CO_2_ (with no adding to complement were also made and referred as the black control). After incubation, cell culture mediums were centrifuged at 3000×*g* for 5 min and the absorbance of the supernatant was measured at 413 nm, the plague forming cell contents were expressed as optical density (OD).

### Determination of half hemolytic value (HC_50_)

The serum hemolysin level was determined according to the method of Yu et al. (2014), with some modification. Briefly, six mice were randomly selected from each group. Blood samples were collected after 24 h of the last drug administration and centrifuged at 2000×*g* for 10 min. The serum was diluted 100 times with stroke-physiological saline. Exactly 0.5 mL of 10% SRBCs and 1 mL of fresh guinea pig serum (1:10 dilution) was added to the reaction tubes filled with 1 mL of diluted serum samples. At the same time, the blank control was made with 1 mL of physiological saline instead of mouse serum. After incubation for 10 min at 37 °C, the reaction tubes were immediately transferred to an ice bath and centrifuged at 2000×*g* for 10 min. Then, 1 mL of the supernatant was mixed with 3 mL of Drabkin’s solution at 37 °C and reacted for 10 min, at which point the absorbance was measured at 540 nm. The absorbance of SRBC half hemolytic value (HC_50_) was also measured with 0.25 mL of 10% SRBCs and 4 mL Drabkin’s solution in the same way as above. The HC_50_ was calculated using following equation:

$${\text{HC}}_{50} = \frac{\text{Sample OD value}}{{ {\text{Sheep erythrocyte OD value}}}} \times {\text{Diluted times}}$$

### Determination of IL-2, IL-6, TNF-α, IgG, and IgM in serum

Whole blood was obtained from BALB/c mice killed under sterile conditions and centrifuged at 1000×*g* and 4 °C for 20 min, while the upper layer contained the serum. The amounts of IL-2, IL-6, TNF-α, IgG, and IgM in the serum were determined using an ELISA kit (Chenglin, Beijing) according to the instructions of the manufacturer.

### Statistical analysis

Statistical values were expressed as mean ± standard deviations (SD) and analyzed using the SPSS software program (version 20.0; IBM Corp., Armonk, NY, USA). The data was subjected to one-way analysis of variance (ANOVA) for mean comparison, and the significant differences were detected by the Duncan`s test, with *p *< 0.05 indicating the difference was significant.

## Results and discussion

### Proximate compositions and oligopeptide yield of raw and fermented cottonseed meal

The nutritional compositions of cottonseed meal and cottonseed meal oligopeptide were shown in Table 1. As compared with the raw cottonseed meal, the crude protein and reducing sugar content in the cottonseed meal oligopeptide samples were increased (*p *< 0.05), which were 1.95 and 7.85 times higher, respectively. Additionally, the content of ash was decreased by 80.72%, while crude fiber, fat, and free gossypol were not detected in cottonseed meal oligopeptide samples. As compared with the raw cottonseed meal, cottonseed meal oligopeptide showed an increase in crude protein and reducing sugar content, while the amounts of other compounds such as crude fat, fiber ash and free gossypol showed a decrease trends (Table 1).

**Table 1:** Average oligopeptide yield and composition of cottonseed meal and cottonseed meal oligopeptide

Compositions	Cottonseed meal (%)	Cottonseed meal oligopeptide (%)
Oligopeptide yield	2.78 ± 0.21	24.4 ± 1.28^a^
Protein	46.33 ± 0.15	90.56 ± 0.18
Fiber	10.37 ± 0.31	ND
Fat	0.46 ± 0.08	ND
Ash	6.58 ± 0.03	1.27 ± 0.06
Free gossypol	634.01 ± 19.90	ND
Reducing sugar	0.40 ± 0.06	3.14 ± 0.09

*ND* not detected

^a^This refers to the production of oligopeptide in the fermentation of cottonseed meal

The oligopeptide yields of raw and fermented cottonseed meal were also evaluated, with results showing that, as compared with the 2.78% of oligopeptide yield from the raw cottonseed meal, the oligopeptide yield increased to 24.47% in the fermented cottonseed meal, and indicating that solid-state fermentation was a good way to improve the oligopeptide contents in cottonseed meal. Similar results were also reported by Sun et al. (2015), who also found an increasing yield of peptides to be present in cottonseed meal after solid-state fermentation. He et al. (2012) reported a significant increase in the crude protein content of peptides after water extraction from rapeseed meal. Similarly, Zhang et al. (2011) evaluated the protein content in the fermented peanut meal and stated that the crude protein increased to 96.07% after fermentation. This is probably attributable to the degradation of macromolecule protein by microbial fermentation or enzymolysis, the protein degraded into peptide, which is more soluble in water than fat and fiber, and eventually increasing the content of protein in fermentation broth.

Although there were a large number of researches into the protein isolation and the enzymes of cottonseed meal, the detection of reducing sugar in extracted cottonseed meal or cottonseed meal peptides has not been reported yet (Gao et al., 2010; Sun et al., 2015). In the present study, the reducing sugar content in cottonseed meal oligopeptide was 3.14%, which was 7.85 times higher than the raw cottonseed meal. And this result might attribute to the addition of sucrose as a carbon source for microbial growth in the solid fermentation medium. During the fermenting process, the sucrose was degraded into glucose by microorganism and remained in the matrix. Additionally, our study also found a lower content of stachyose and raffinose in the fermented cottonseed meal than the raw one.

### Molecular weight distribution of cottonseed meal oligopeptide

**Fig. 1:**
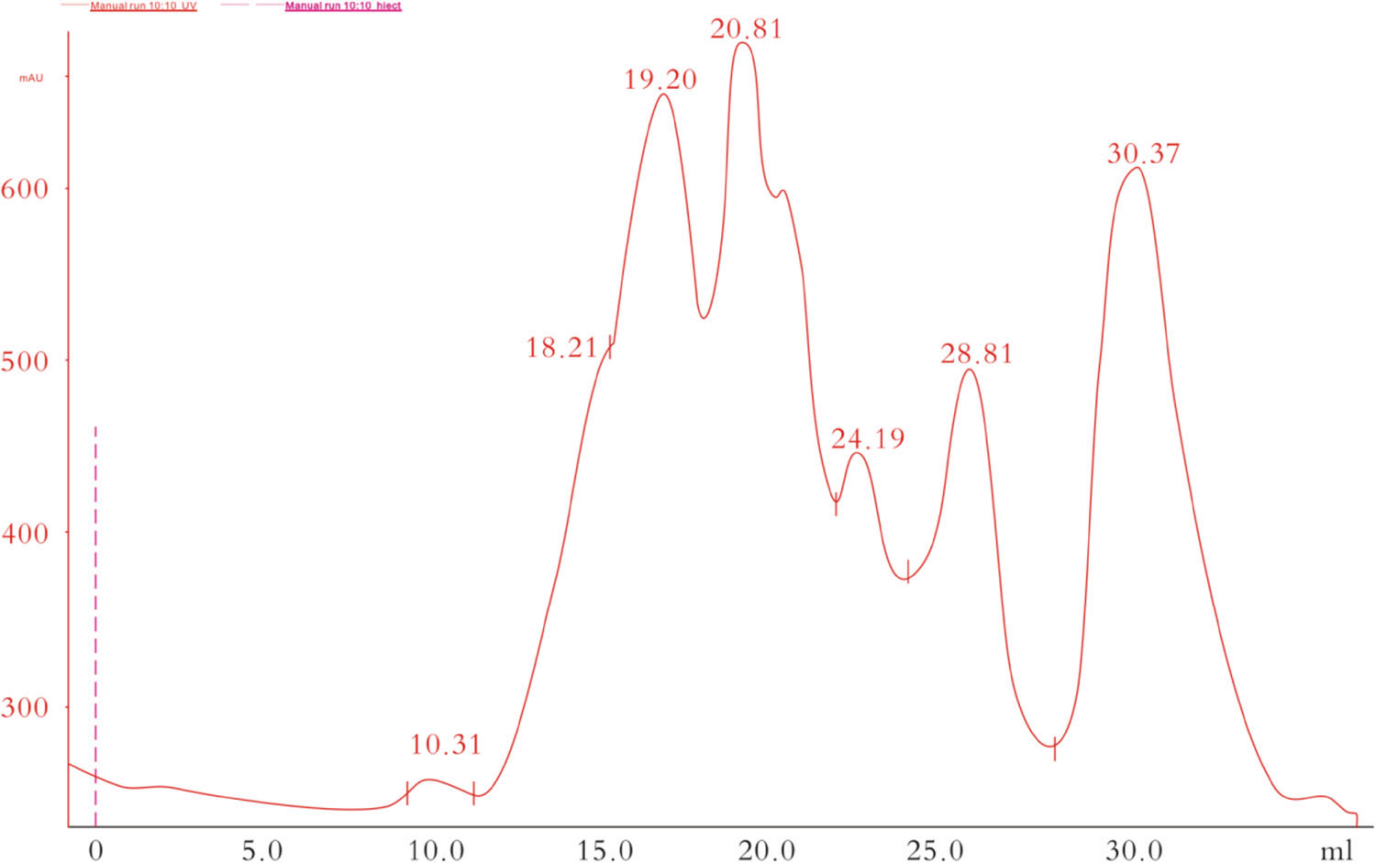
Molecular weight distribution of oligopeptide derived from solid-fermented cottonseed meal. x-axis, elution volume (mL); y-axis, response value (mAU)

As shown in Fig. 1, the molecular weight of cottonseed meal oligopeptide was continuously distributed over the time, which was automatic analyzed with integral software. The distribution was divided into 4 molecular weight intervals, of which 89.96% of the peptide molecular weight was less than 623 Da. However, in the previous studies performed by Sun et al. (2015), the authors reported that the molecular weight distribution profile of peptides mainly ranged from less than 158 to 3000 Da, with 56.52% of the fractions being less than 1000 Da. This discrepancy might attribute to the differences in strains and fermentation substrates.

### Amino acid composition of cottonseed meal oligopeptide

The amino acid composition of cottonseed meal oligopeptide is shown in Table 2. The total amounts of amino acids in solid-state fermented cottonseed meal oligopeptide were about 77.92%. Glutamic acid, aspartic acid, and arginine were the main amino acids, and accounted for 25.84, 8.14, and 7.09%, respectively. The total amount of acidic amino acids was 33.98% in the oligonucleotide of solid-state fermented cottonseed meal, accounting for 43.6% of the total amino acids, while the total amount of basic amino acids was 10.78%, accounting for 13.8% of the total amino acids.

**Table 2:** Composition of total amino acid in cottonseed meal oligopeptide

Amino acids	Content (%)
Aspartic acid	8.14 ± 0.12
Threonine	1.73 ± 0.03
Serine	2.66 ± 0.06
Glutamic acid	25.84 ± 0.54
Glycine	2.73 ± 0.07
Alanine	2.18 ± 0.04
Cysteine	2.29 ± 0.06
Valine	3.73 ± 0.10
Methionine	0.81 ± 0.01
Isoleucine	1.60 ± 0.02
Leucine	3.51 ± 0.11
Tyrosine	2.99 ± 0.03
Phenylalanine	5.65 ± 0.09
Lysine	2.50 ± 0.06
Histidine	1.19 ± 0.02
Arginine	7.09 ± 0.18
Proline	3.28 ± 0.05
Total amino acids	77.92 ± 0.81
AAA	33.98 ± 0.33
BAA	10.78 ± 0.14

*AAA* acidic amino acid (e.g., glutamic acid and aspartic acid), *BAA* basic amino acid (e.g., arginine, histidine, and lysine)

### Effects of cottonseed meal oligopeptide on the spleen and thymus indices in cyclophosphamide-treated mice

It is well-known that cyclophosphamide is an important immunosuppressive drug used in clinical studies (Wang et al., 2013) and is capable of damaging the structure of DNA, killing immune cells, interfering with the proliferation and differentiation of macrophages of T- and B-cells, and restraining the humoral and cellular immune response (Xu and Zhang, 2015). Animals treated with cyclophosphamide have been widely used to evaluate the immune regulatory properties of various bioactive components and drugs (Huang et al., 2013). In the present study, the protective effects of cottonseed meal oligopeptide against cyclophosphamide induced immunosuppression were determined by organ indices analysis of spleen and thymus. The effects of cottonseed meal oligopeptide on immune organ index in immunosuppressed mice are described in Table 3. The injection of cyclophosphamide induced a significantly decrease in the thymus and spleen index of mice (*p *< 0.05), indicating that cyclophosphamide could inhibit the growth and development of immune organs in mice significantly. Immune function and immune prognosis are reflected well by the thymus and spleen indices because of the important roles of those organs in nonspecific immunity (Chandrashekar and Venkatesh, 2012). They are the sites of growth and proliferation of immune cells. The thymus and spleen are the two major lymphoid organs severely affected during cyclophosphamide induced immunosuppression (Pang et al., 2007). The destruction of splenocytes and thymocytes were observed in the histopathological sections of cyclophosphamide treated mice (Raj and Gothandam, 2015).

**Table 3:** Effects of oligopeptide on the mouse spleen and thymus indices in cyclophosphamide-treated mice

Group	Thymus index (mg/g)	Spleen index (mg/g)
NC	1.95 ± 0.16^a^	8.17 ± 0.87^a^
CY	1.28 ± 0.15^b^	5.54 ± 0.10^b^
CP-5	1.60 ± 0.25^a^	7.32 ± 0.91^a^
CP-10	1.99 ± 0.20^a^	9.25 ± 0.11^a^
CP-20	1.85 ± 0.19^a^	8.57 ± 1.43^a^

Different characters in each column indicate significantly different at 0.05 levels

*NC* normal control, *CY* cyclophosphamide model control, *CP* cottonseed meal oligopeptides were administered via gavage in mice by cyclophosphamide treated

However, after intake of oligopeptide, the thymus and spleen index showed an increased vale trends and the indexes in the 10 mg/mL (CP-10) group was much higher than that in the 5 mg/mL (CP-5) and 20 mg/mL (CP-20) groups, though no significant differences were observed (*p *> 0.05). The results of the experiment showed significant increases in the thymus and spleen indices in the oligopeptide groups versus in the cyclophosphamide-treated group. In particular, oligopeptide treatment restored the spleen index to a level near or above that in the normal control group. The treatment with oligopeptide protects the immune organs against the damage caused by cyclophosphamide and the protective effect of oligopeptide might be due to reversing the immunosuppression caused by cyclophosphamide treatment. Similar results were also reported, in that some other macromolecules could also improve the immune organ indices of mice and inhibit atrophy of immune organs significantly (Kumar and Venkatesh, 2016).

### Effects of cottonseed meal oligopeptide on the PFC and HC_50_ in cyclophosphamide-treated mice

The effects of cottonseed meal oligopeptide on the (PFC) and (HC_50_) are shown in Table 4. Notably, the numbers of PFC and HC_50_ in the CY group were significantly lower than in the NC group (*p *< 0.05). Intake of oligopeptide in different concentrations could increase the number of PFC in immunosuppressed mice and even return them to normal levels. When the concentration of oligopeptide was increased to 10 and 20 mg/mL, the PFC was even much higher than that of the control group, but the difference was not significant (*p *> 0.05). Intake of oligopeptide at a low concentration also increased the HC_50_ in mice, but the achieved level remained a little lower than that which was normal. However, the middle concentration of oligopeptide (10 mg/mL) increased the HC_50_ significantly to a similar level as that seen in normal mice. However, with the increasing concentration of oligopeptide (20 mg/mL), the PFC and HC_50_ showed a decrease trend.

**Table 4:** Effects of oligopeptide on the PFC and HC_50_ in cyclophosphamide-treated mice

Group	PFC	HC_50_
NC	0.36 ± 0.01^a^	7.65 ± 0.39^a^
CY	0.28 ± 0.04^b^	5.38 ± 0.49^b^
CP-5	0.37 ± 0.01^a^	6.77 ± 0.45^b^
CP-10	0.40 ± 0.02^a^	7.97 ± 0.20^a^
CP-20	0.39 ± 0.02^a^	7.84 ± 0.41^a^

Different characters in each column indicate significantly different at 0.05 levels

*NC* normal control, *CY* cyclophosphamide model control, *CP* cottonseed meal oligopeptides were administered via gavage in mice by cyclophosphamide treated, *PFC* plague forming cell, *HC*_50_ half hemolytic value

An increase in the number of plague forming cell against a particular antigen indirectly signifies the stimulatory effect on the humoral immunity (Bilal et al., 2003). The formation of serum hemolysin with SRBC immunization reflects humoral immunologic function (Chen et al., 2012). In the present study, the treatment of oligopeptide significantly increased the level of HC_50_ and the number of PFC in mice spleens. These findings indicate that oligopeptide significantly stimulated the humoral immunity by enhancing the antibody produced against the SRBC and increasing the level of serum hemolysis. The present study revealed that humoral immunity played a vital role in the immune-stimulation achieved by oligopeptide.

### Effects of cottonseed meal oligopeptide on serum IL-2, IL-6, and TNF-α level in cyclophosphamide-treated mice

The effects of oligopeptide on the level of IL-2, IL-6, and TNF-α are shown in Table 5. As compared with the control group, the levels of IL-2 and IL-6 in the CY group were significantly lower (*p *< 0.05), but no difference was seen with regard to TNF-α (*p *> 0.05). Intake of oligopeptide at different concentrations may promote the recovery of the immune system in mice and help return the IL-2 content back to the normal level. For IL-6, a low concentration of oligopeptide (5 mg/mL) could also increase the content, but the effect was not significant (*p *> 0.05), while the medium (10 mg/mL) and high concentrations (20 mg/mL) of cottonseed meal oligopeptide may return the serum IL-6 content in mice to the normal level. For TNF-α, different concentrations of oligopeptide could increase the content of TNF-α in the serum of immunosuppressed mice, and the increase was closely associated with the concentrations, the TNF-α in the group of high oligopeptide (20 mg/mL) was significantly different from that in the control group (*p *< 0.05).

**Table 5:** Effects of oligopeptide on serum IL-2, IL-6, TNF-α, IgG, and IgM in cyclophosphamide-treated mice

Group	IL-2 (pg/mL)	IL-6 (pg/mL)	TNF-α (ng/L)	IgG (µg/mL)	IgM (µg/mL)
NC	1739 ± 22.3^a^	79.74 ± 2.78^a^	595.7 ± 23.9^a^	382.9 ± 27.9^a^	50.93 ± 2.00^a^
CY	1653 ± 41.0^b^	69.46 ± 2.68^b^	564.5 ± 20.8^a^	317.4 ± 21.5^b^	32.32 ± 3.67^b^
CP-5	2101 ± 17.4^a^	72.25 ± 4.03^b^	589.5 ± 10.6^a^	337.8 ± 10.1^a^	35.20 ± 1.97^b^
CP-10	2106 ± 18.1^a^	84.56 ± 4.80^a^	600.0 ± 37.9^a^	439.1 ± 42.9^c^	52.53 ± 2.9^a^
CP-20	2047 ± 10.0^a^	82.53 ± 5.92^a^	685.8 ± 56.8^b^	396.9 ± 16.3^ac^	43.56 ± 1.64^a^

Different characters in each column indicate significantly different at 0.05 levels

*NC* normal control, *CY* cyclophosphamide model control, *CP* cottonseed meal oligopeptides were administered via gavage in mice by cyclophosphamide treated

Cytokines are high-activity, small-molecule proteins (e.g. IL-2, IL-6, TNF-α,) secreted by cells that have many functions. They play important roles in the differentiation and development of immune cells, the immune response, immune regulation, inflammatory response, and hematopoietic function. For instance, IL-2 is an important cytokine produced by activated T-cells, and is necessary for the growth, proliferation, and differentiation of T-cells to become effector T-cells (Yu et al., 2014), while IL-6 is also considered as a major immune and inflammatory mediator that can promote the proliferation and differentiation of B-cells and T-cells, and induce antibodies secreted from mature B-cells (Song et al., 2002); TNF-α, mainly by activating monocytes and macrophages, can kill and inhibit tumor cells; promote neutrophil phagocytosis; assist with anti-infection initiatives; lower fever; coax myeloid leukemia cells into macrophage differentiation; and prompt cell proliferation and differentiation, which is an important inflammatory factor (Kumar et al., 2011). Early studies have reported that different nutritional components could modulate the immune system by stimulating the expression/production of pro-inflammatory cytokines and anti-inflammatory cytokines (Kau et al., 2011). In this study, the activation of innate and adaptive immune responses was measured by various cytokines and the levels of IL-2, IL-6, and TNF-α were found to be significantly higher in the oligopeptide groups than in the cyclophosphamide-treated group, suggesting that oligopeptide promotes immune cell proliferation and differentiation. Similar findings have been reported by Yoshikawa et al. (1993), who found that soybean peptides can induce TNF-α in mouse and thus play a role in immune regulation.

### Effects of cottonseed meal oligopeptide on serum IgG and IgM contents in cyclophosphamide-treated mice

IgG and IgM are the major immunoglobulins involved in the complement activation, opsonization, and neutralization of toxins (Miller et al., 1991). The effects of oligopeptide on IgG and IgM in immunosuppressed mice are shown in Table 5. In comparison with the control group, the injection of cyclophosphamide reduced the serum IgG and IgM levels significantly (*p *< 0.05). After intake of oligopeptide (5 mg/mL), the IgG content in immunosuppressed mice increased significantly, back to the normal level. However, the IgM content following cottonseed meal oligopeptide intake showed no significant difference. The middle concentrations of oligopeptide (10 mg/mL) increased the IgG and IgM in immunosuppressed mice significantly (*p *< 0.05). In particular, the IgG content was significantly higher than in the control group (*p *< 0.05). However, intake of high concentration of oligopeptide showed little effect on both of the IgG and IgM in immunosuppressed mice. Our results demonstrated that oligopeptide significantly increased the levels of IgG and IgM in cyclophosphamide-treated immunosuppressed mice. This suggests that the intake of oligopeptide can enhance humoral immunity effectively.

In conclusion, the oligopeptide derived from cottonseed meal by solid-state fermentation had a protective and restorative effect against cyclophosphamide induced immunosuppression in mice. The use of oligopeptide may be effective to enhance systemic immune responses or to modulate the functions of immune-competent cells. These effects are very important in immune-compromised hosts. Oligopeptide derived from fermented cottonseed meal should be further investigated for possible immune-stimulatory applications in the food and pharmaceutical industries.
